# pH-Sensitive Poly(β-amino ester)s Nanocarriers Facilitate the Inhibition of Drug Resistance in Breast Cancer Cells

**DOI:** 10.3390/nano8110952

**Published:** 2018-11-19

**Authors:** Mengxue Zhou, Xingcai Zhang, Jin Xie, Rongxiang Qi, Huiru Lu, Stefano Leporatti, Jun Chen, Yi Hu

**Affiliations:** 1 CAS Key Laboratory for Biomedical Effects of Nanomaterials and Nanosafety, Multidisciplinary Research Division, Institute of High Energy Physics, Chinese Academy of Sciences, Beijing 100049, China; zhoumx@ihep.ac.cn (M.Z.); xiejin5739@163.com (J.X.); qrx198913@163.com (R.Q.); luhuiru@ihep.ac.cn (H.L.); 2 University of Chinese Academy of Sciences, Beijing 100049, China; 3 John A. Paulson School of Engineering and Applied Sciences, Harvard University, Cambridge, MA 02138, USA; xingcai@mit.edu; 4 CNR Nanotec-Istituto di Nanotecnologia, Polo di Nanotecnologia, 73100 Lecce, Italy

**Keywords:** nanoparticles, pH responsive, poly(β-amino ester)s, doxorubicin, multidrug resistance

## Abstract

Multidrug resistance (MDR) remains an unmet challenge in chemotherapy. Stimuli-responsive nanocarriers emerge as a promising tool to overcome MDR. Herein, pH-sensitive poly(β-amino ester)s polymers (PHP)-based micellar nanoparticles were synthesized for enhanced doxorubicin (DOX) delivery in drug resistant breast cancer MCF-7/ADR cells. DOX-loaded PHP micelles showed rapid cell-internalization and lysosomal escape in MCF-7/ADR cells. The cytotoxicity assays showed relatively higher cell inhibition of DOX-loaded PHP micelles than that of free DOX against MCF-7/ADR cells. Further mechanistic studies showed that PHP micelles were able to inhibit P-glycoprotein (P-gp) activity by lowering mitochondrial membrane potentials and ATP levels. These results suggested that the enhanced antitumor effect might be attributed to PHP-mediated lysosomal escape and drug efflux inhibition. Therefore, PHP would be a promising pH-responsive nanocarrier for enhanced intracellular drug delivery and overcoming MDR in cancer cells.

## 1. Introduction

Multidrug resistance (MDR) is a common issue in chemotherapy, resulting in the poor prognosis. Therefore, development of effective approaches to overcome the drug resistance remains an unmet challenge [1,2,3]. It has been reported that several mechanisms are associated with MDR and one of them is P-glycoprotein (P-gp)-mediated efflux of intracellular drugs and consequently compromised therapeutic efficacy [4]. To address this issue, an increasing number of studies have exploited stimuli-responsive nanoparticle-based drug delivery systems to reverse P-gp-mediated MDR [5,6,7].

Stimuli-responsive nanoparticles can change their physicochemical properties such as size, morphology, surface property in presence of specific stimuli [8]. The characteristic low pH values of tumor site are one of the widely used endogenous stimuli [9,10,11]. Due to the fast proliferation feature of tumors, a severe deficit in both nutrients and oxygen renders tumor cells to take an alternative glucose metabolism, leading to the enrichment of acidic metabolites. As a result, the extracellular pH values in tumor area are typically in a range from 6.0 to 7.0 [12]. Moreover, when the nanoparticles are taken up by cells through the endocytosis pathway, rapid acidification is initiated in endo/lysosomes, where the pH values can reach as low as 4.5–6.0. Such pH changes can be exploited to initiate the change of nanoparticles, such as the size reduction, disassembly or aggregation to control the delivery of drugs to tumors [13,14,15]. To obtain such pH-responsive property, one strategy is the use of polymers containing ionizable groups that undergo hydrophilicity and hydrophobicity switches via protonation or deprotonation [16]. As a type of classical ionizable polymers, poly(β-amino ester)s have been extensively explored as biodegradable and biocompatible materials for anticancer drug delivery [17,18,19]. It generally has a pKb value of ~6.5 from its tertiary amine groups, which is tunable by modulating the adjacent functional groups [20]. On the other hand, the rapid protonation of poly(β-amino ester)s-based nanoparticles upon acidic pH can induce “proton sponge” effects, which facilitates the escape of encapsulated drugs from lysosomes.

**Scheme 1:**
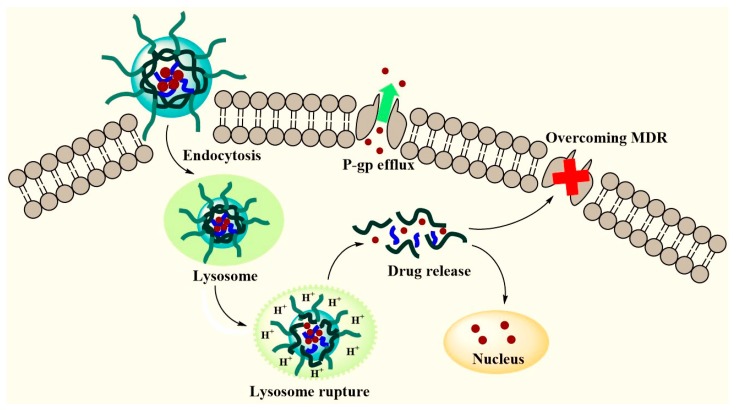
Schematic illustration of DOX-loaded pH-sensitive Poly(β-amino ester)s (PHP) nanoparticles to overcome multidrug resistance (MDR).

Herein, pH-sensitive polymer poly(β-amino ester)s (PHP) were synthesized via Michael addition polymerization. The PHP polymeric micelles, d-alpha-tocopherol polyethylene glycol 1000 succinate (TPGS) micelles and PHP/TPGS mixed micelles were prepared by dialysis against PBS buffer (pH = 7.4) from organic solution. The buffer capacity of PHP micelles was examined in the pH range of 3–7.5. The encapsulated doxorubicin (DOX) was released in a higher rate from PHP-containing micelles in a weakly acidic condition (pH 6.5) than that at pH 7.4. Confocal microscopy was used to investigate the intracellular drug delivery and translocation in MCF-7 cells and MCF-7/ADR cells. The amine groups in PHP segments facilitated translocation of DOX from lysosomes to the cytosol. Moreover, the effect of the micelles on the P-gp activity, intracellular ATP levels and mitochondrial membrane potentials were also investigated. We found that PHP-based nanomedicine could potently reverse the drug resistance in MCF-7/ADR cells. Taken together, these results revealed a promising potential of PHP copolymer to be a pH-responsive and P-gp inhibitory nanocarrier for chemotherapy (Scheme 1).

## 2. Materials and Methods

### 2.1. Synthesis and Characterization of PHP Copolymer

The pH-sensitive polymers were synthesized as follows: 1,6-hexanediol diacrylate (HDDA, 226 mg, 1.0 mmol), 4,4′-trimethylene dipiperidine (TMDP, 252 mg, 1.2 mmol) and methoxy PEG acrylate (500 mg, 0.1 mmol) were mixed in a 20-mL vial with 4 mL DMSO and 2 mL chloroform for 3 days at 50 °C with the protection of argon. The mixture was then precipitated in diethyl ether twice, followed by being dried in vacuum. ^1^H NMR studies were carried out by using a Bruker AMX 500 NMR spectrophotometer (500 MHz) with CDCl_3_ as the solvent. ^1^H NMR (ppm): *δ* 1.20–1.40 (10H, –(H*C**H***)_2_CH(C***H*_2_**)_3_CH(H*C**H***)_2_–), 1.40 (4H, –(***H****C*H)_2_CH(CH_2_)_3_CH(H*C**H***)_2_–), 1.65–1.70 (6H, –OCH_2_CH_2_(C***H*_2_**)_2_CH_2_CH_2_O– and –(CH_2_)_2_C***H*(**CH_2_)_3_C***H***(CH_2_)_2_–), 1.90–2.10 (4H, –COOCH_2_C***H*_2_**(CH_2_)_2_C***H*_2_**CH_2_COO–), 2.50 (4H, –C***H*_2_**COO–), 2.65 (4H, –(HC***H***)_2_N–), 2.80 (4H, –(HC***H***)_2_N–), 3.3–3.4 (3H, C***H*_3_**O–), 3.5–3.7 (4XH, C***H*_2_** in PEG repeat unit), 4.0–4.1 (4H, –COOC***H*_2_**–), 4.20 (2H, –COOC***H*_2_**–).

### 2.2. Preparation and Characterization of Micelles

The PHP micelles with or without TPGS were prepared by the dialysis method. Briefly, PHP copolymer (10 mg) and TPGS1k (10 mg) were dissolved in 2 mL of DMF. Subsequently, 10 mL of PBS buffer (pH = 7.4) was added dropwise and vigorously stirred for 2 h. The solution was then dialyzed in PBS buffer (pH = 7.4) for 24 h (molecular weight cut-off = 7000). The buffer capacity of the polymers was analyzed by titration with 0.1 M HCl.

Buffer Capacity (%) = *V*(HCl) × 0.1 M/*N* mol × 100%

where *V* is volume of the 0.1 M HCl and *N* is the total amount of amine groups in polymer solution.

Hydrodynamic diameter and size of the nanocarriers were examined with Zetasizer Nano-S from Malvern Instruments. The stability of nanoparticles was examined as previously described [21]. The transmission electron microscopy (TEM) analysis was conducted as the following. A drop of the micelle solution was drop cast on a carbon-coated copper grid and dried by filter paper. Then 2% (*w*/*v*) of uranyl acetate solution was dropped on the grid. A 1-min contact was allowed before removal of excess liquid with filter paper. The grid was air-dried for 1 h before observation under the microscope. The average diameters of particles were measured from 10 particles in TEM micrographs.

### 2.3. Preparation and Characterization of DOX-Loaded Copolymer Micelles

DOX-loaded micelles of PHP, TPGS and PHP/TPGS copolymer were prepared as follows: 2 mg DOX dissolved in mixture of 0.1 mL of DMSO and 0.9 mL chloroform were stirred with 1.5 equiv triethylamine for 1 h. 10 mg PHP amphiphilic copolymers were then added into the desalinated DOX solution and allowed for the complete dissolution. Then 10 mL PBS buffer (pH = 7.4) was dropwise added along with ultrasonication and emulsified for another 10 min by pulsed sonication. The emulsion was then stirred at room temperature overnight to allow evaporation of the chloroform. The solution was then dialyzed in PBS buffer (0.01 M, pH = 7.4) for 24 h (molecular weight cut-off = 3500). The drug loading efficiency (the ratio of loaded drug (weight) to feeding drug (weight)) was determined by measurement of its absorbance at 480 nm. To analyze the drug loading content (the proportion of loaded drug (weight) in total weight of nanoparticles), a calibration curve was used with different DOX concentrations.

$$\mathrm{Drug}-\text{loading content}\;\left(\%\right)=\frac{\text{weight of loaded drug}}{\text{weight of copolymer}+\text{weight of loaded drug}}\times 100\%$$

$$\mathrm{Drug}-\text{loading efficiency}\;\left(\%\right)=\frac{\text{weight of loaded drug}}{\text{weight of feeding drug}}\times 100\%$$

### 2.4. pH-Sensitive Release of DOX from Micelles

DOX release was examined as the following. The sample containing 3 mL DOX-loaded polymeric micelles was placed in a dialysis bag (molecular weight cut-off = 3.5 kDa). The dialysis bag was placed in a vial with 17 mL PBS (0.01 M, pH = 7.4, 6.5 or 5.0) at 37 °C and stirred at 200 rpm. At different time intervals, 5 mL solution was withdrawn from the vial to measure DOX absorbance at 480 nm and 5 mL of fresh PBS was added to refill the vial. The concentrations of DOX were calculated according to a standard curve of DOX absorbance. Similarly, DOX release in cell culture media was also examined.

### 2.5. Cytotoxicity Assays

MCF-7 cells were obtained from the Bogoo Biotech. Inc. (Shanghai, China) and grown in Dulbecco’s Modified Eagle’s Medium (DMEM, HyClone™) with 10% fetal bovine serum (FBS, GIBCO), 1.0 × 10^5^ U/L penicillin and 0.1 mg/mL streptomycin (Sigma, Bejing, China). MCF-7/ADR cell line was obtained from the Cell Center, Cancer Hospital, Chinese Academy of Medical Sciences (Beijing, China). Cells were cultured in Roswell Park Memorial Institute (RPMI) medium with 10% FBS, 1.0 × 10^5^ U/L penicillin and 0.1 mg/mL streptomycin (Sigma, Bejing, China) at 37 °C with 5% CO_2_.

The cytotoxicity of the nanoparticles was analyzed by CCK-8 assay. MCF-7 and MCF-7/ADR cells were seeded into a 96-well plate at a density of 8000 cells per well. After 24 h, the growth medium was replaced with fresh media. Afterward, the nanoparticles were added into wells (six wells per sample). After a predetermined time, the culture medium was removed. 10% CCK-8 in DMEM or RPMI was added into each well and incubated for another 0.5 h. The absorbance of each well was measured at a test wavelength of 450 nm. The cell viability was calculated as

$$\text{cell viability}\;\left(\%\right)=\frac{I_{sample}-I_{blank}}{I_{control}-I_{blank}}\times 100\%$$

### 2.6. Cellular Uptake and Intracellular Translocation of Nanoparticles

The cells were seeded in a 6-well plate. After the cells reached 70−80% confluence, the cells were incubated with free DOX or DOX-loaded nanoparticles for 2 h or 24 h at 37 °C. Afterward, the cover slip was washed three times with pre-warm PBS and cells were fixed by 4% paraformaldehyde in PBS at room temperature for 15 min. After fixation, the cells were incubated with PBS containing 0.1% Triton X-100 for 10 min and then rinsed with PBS three times. The cells were stained with 10 mM Hoechst 33342 for 20 min and then rinsed with PBS three times. The cells were imaged by a confocal laser scanning microscope.

### 2.7. Rhodamine 123 (Rh123) Accumulation and Efflux

P-gp activity assays were carried out in MCF-7/ADR cells. TPGS was used as a standard P-gp inhibitor. Rh123, a P-gp substrate, was used to indicate the transport activity of P-gp [22,23]. Briefly, MCF-7/ADR cells were seeded in a 6-well plate and incubated overnight. The cells were incubated with Rh123 (5 μg/mL), together with PHP or TPGS (50 μg/mL). After 2-h incubation, the cells were washed three times with PBS. The fluorescence of Rh123 was examined by fluorescence-activated cell sorting (FACS) using ModFit software.

For Rh123 efflux assay, cells were preincubated with 5 μg/mL Rh123 for 1 h, followed by incubation with the micelles for another 1 h. Afterward, the cells were washed and analyzed as described above.

### 2.8. Mitochondrial Membrane Potential

The probe 5,5′,6,6′-tetrachloro-1,1′,3,3′-tetraethylbenzimidazolylcarbocyanine iodide (JC-1) can transform from a monomer (red fluorescence) to an aggregated form (green fluorescence) depending on mitochondrial membrane potential [24]. MCF-7/ADR cells were cultured in 6-well plates (1 × 10^6^ cells/well) for 24 h, followed by the incubation with the micelles for 2 h. The cells were then stained with 500 μL of JC-1 (10 μg/mL) working solution for another 20 min. After washing twice, the cells were examined with a multi-mode microplate reader (Infinite 200 Pro, Tecan, Zürich, Switzerland).

### 2.9. Intracellular ATP Level Assay

MCF-7/ADR cells were cultured in 6-well plates (1 × 10^6^ cells per well) for 24 h at 37 °C, followed by the incubation with the micelles for 4 h. ATP levels were examined using the ATP assay kit (Beyotime Institute of Biotechnology, Jiangsu, China) and analyzed by a multi-mode microplate reader (Spark™ 10M, Tecan, Zürich, Switzerland). The ATP levels were determined according to the ATP standard curve.

### 2.10. Lactate Dehydrogenase (LDH) Assay

MCF-7/ADR cells (8 × 10^3^ cells/well) were seeded in a 96-well plate and incubated overnight in 100 μL of RPMI medium containing 10% FBS. Afterward, the cell culture medium was replaced with the fresh medium containing 100 μL of PHP polymer solutions with various concentrations at pH 7.4 or pH 5. After the incubation at 37 °C for 1.5 h, the plates were centrifuged at 180 g. 80 μL of aliquots were collected from each well for LDH assay (Beyotime Institute of Biotechnology, Jiangsu, China) according to the manufacturer’s protocol.

### 2.11. Statistics

For quantitative analysis of DOX fluorescence in cells and the cellular effects of PHP, ANOVA with post-hoc Tukey’s test by SPSS was applied to analyze the statistical difference. Other studies were analyzed by two-tailed student’s *t*-test in Excel. *p* < 0.05 was accepted as a statistically significant difference.

## 3. Results

### 3.1. Synthesis of PHP Copolymer

**Figure 1:**
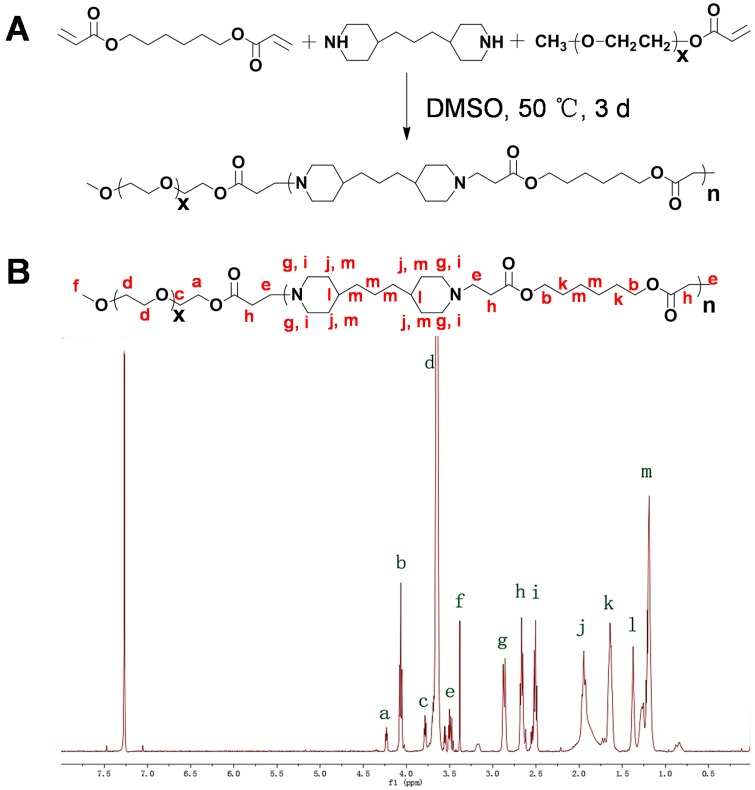
Synthesis of pH-sensitive PHP copolymer (**A**) and its ^1^H NMR spectrum recorded on a Bruker AMX 500 NMR spectrophotometer (500 MHz) (**B**).

The structures of the copolymers are shown in Figure 1. PHP copolymers were prepared through Michael addition-type polymerization according to the methods in a previous report [25]. The chemical structure of copolymers was characterized by ^1^H NMR. In the polymerization, we fed excess of diamine monomer to assure the complete consumption of acrylate groups. For the convenience of calculating average number molecular weight (Mn) of the products, excess of diacrylate monomers were added to ensure the appearance of acrylate groups at one end of the polymers post the reaction. The integrals of peaks (*δ* = 5.8–6.2) ascribed to the end groups of acrylates in the purified polymers, peaks (*δ* = ~3.7) ascribed to the PEG moiety and peaks (*δ* = ~4.1) ascribed to the methylene groups adjacent to the ester bonds were used to calculate the Mn of copolymer (Figure 1). As a result, there were about 6 repeat units in the poly(β-amino ester)s segment, indicating the calculated Mn of copolymer was ~8000. These results showed a successful preparation of PHP block-PEG copolymers.

**Figure 2:**
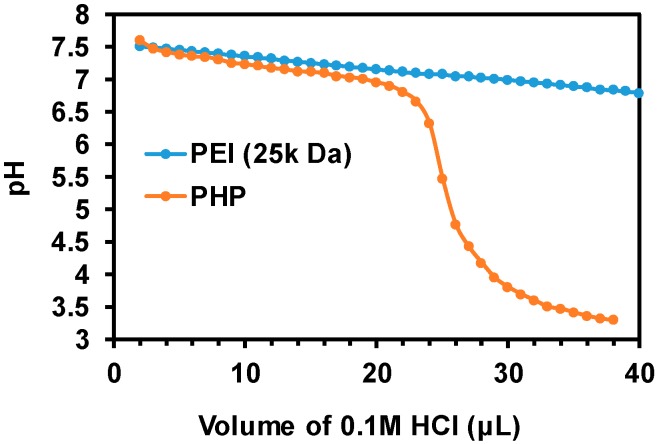
Titration curve of branched polyethylenimine (PEI, Mw = 25 k Da, 2 mg/mL) and PHP (2 mg/mL) by addition of 0.1 M HCl (20 °C).

The endo/lysosomes are one of physical barriers being responsible for the suboptimal effectiveness of intracellular drug delivery. Polycations, for example polylysines, polyethylenimine (PEI), polyamidoamines and so forth, possessed high buffer capacity to enhance the endosomal escape via a strong proton sponge effect. To verify the buffer capacity in vitro, the pH of a polymer solution was examined by adding 0.1 M HCl. The buffer capacity of PHP was in the range of pH 6.5–7.4 (Figure 2 and Figure S1), as previously reported [26]. The results also showed that PHP had a comparative buffer capacity in comparison with that of PEI in the pH range of 6.5–7.4, which was known as a gold standard for non-viral gene vector. Since the existence of abundant amino groups in PEI, it exhibited a high buffer capacity in a wide range of pH values. The polymer chain with the high buffer capacity between pH = 5.5–7.4 could be ionized rapidly, which in turn elevates osmotic pressure of lysosomes until burst. The “proton sponge” effect is important for lysosomal escape and contributes to the efficient intracellular drug delivery.

### 3.2. Characterization of PHP Micelles

**Figure 3:**
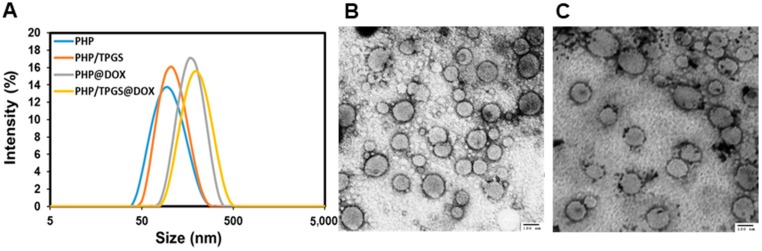
Diameter and size distribution of blank micelles and DOX-loaded micelles by dynamic light scattering (DLS) (**A**) and TEM micrograph of blank PHP micelles (**B**) and PHP/TPGS micelles (**C**). The scale bar is 100 nm.

The copolymer was dissolved in DMSO and the solution was dialyzed in PBS (pH 7.4) for 24 h. Similarly, this method was also applied to prepare TPGS micelles and PHP/TPGS (weight ratio = 1:1)-mixed micelles. The content of drug loading was set at ~33 wt %. The drug loading efficiency was about 88.7%, 98.1%, 83.5%, respectively, for PHP copolymeric micelles, TPGS micelles and PHP/TPGS-mixed micelles. The average diameter of PHP micelles and PHP/TPGS micelles were also shown to be ~100 nm with a monodispersed peak and narrow size distribution (Figure 3A). DOX-loaded PHP micelles were about 200 nm. TEM results showed that the micelles were in a spherical morphology and an average size was about 80 nm and 110 nm for PHP micelles (Figure 3B) and PHP/TPGS micelles (Figure 3C), respectively. These nanoparticles were relatively stable in PBS, 2% BSA, 150 mM sodium chloride or cell culture media (Figure S2).

### 3.3. DOX Release Profiles from the PHP Micelles, TPGS and PHP/TPGS Amphiphilic Copolymer Micelles

**Figure 4:**
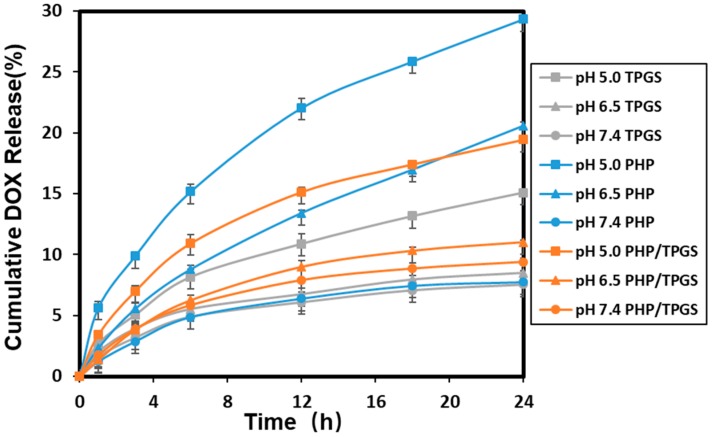
DOX release from PHP micelles, TPGS and PHP/TPGS amphiphilic copolymer micelles at different pH (mean ± SD, *n* = 3).

DOX release profiles were investigated using the dialysis method against different pH buffer solutions, including pH 7.4 corresponding to the normal physiological environment, pH 6.5 for simulating the pH of tumor tissues and pH 5.0 for simulating the pH in late endosomes or lysosomes. As shown in Figure 4, for PHP group, there was a slow release of about 7% of DOX in 24 h at pH 7.4, indicating the stability of the core-shell structure. As expected, the rate of DOX release was higher at pH 6.5 due to the partial protonation of the amine groups in PHP segments. DOX release was fastest at pH 5.0, due to the extensive protonation of PHP segments and subsequent rapid dissociation. For the pH-nonresponsive TPGS micelles, DOX release was in a slow profile. Similar DOX release profiles were observed in cell culture media (Figure S3).

### 3.4. Cellular Uptake and Intracellular Distribution of DOX

**Figure 5:**
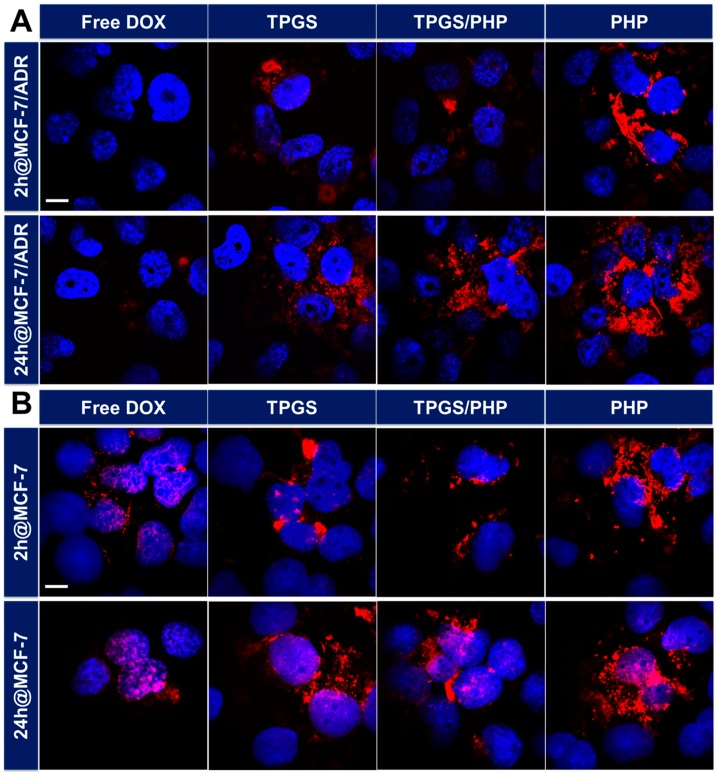

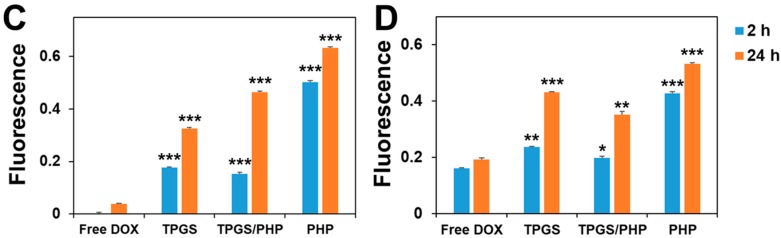
Confocal microscopy images of MCF-7/ADR (**A**) and MCF-7 (**B**) cells incubated with free DOX, DOX-loaded PHP micelles, TPGS micelles and TPGS/PHP micelles for 2 h and 24 h, respectively. Scale bar = 11 µm. Quantitative analysis of fluorescence intensity of DOX in MCF-7/ADR (**C**) and MCF-7 (**D**) cells. Values are mean ± SD, *n* = 6. * *p* < 0.05, ** *p* < 0.01, *** *p* < 0.001.

The cellular uptake and intracellular distribution of DOX were investigated using confocal microscopy in MCF-7 and MCF-7/ADR cells. While MCF-7/ADR cells have a relatively high expression level of P-gp, MCF-7 cells were used a model of low P-gp expression, although these cells might have different genetic backgrounds [27]. In this study, the nuclei were stained by Hoechst 33342. For MCF-7 cells, after the incubation with DOX and DOX-loaded micelles, DOX-loaded PHP micelles first released DOX and DOX was accumulated in or around the nuclei, whereas the fluorescence of free DOX was relatively weaker (Figure 5). These results indicated that DOX-loaded PHP micelles could be taken up fast by the cells, as compared to free DOX. For MCF-7/ADR cells, low DOX fluorescence was observed in free DOX-treated cells. By contrast, remarkably high DOX fluorescence could be seen in cells treated by DOX-loaded PHP micelles. These results suggested that PHP nanocarriers could facilitate intracellular delivery of DOX in drug resistant cancer cells.

### 3.5. Intracellular Trafficking of Nanoparticles

**Figure 6:**
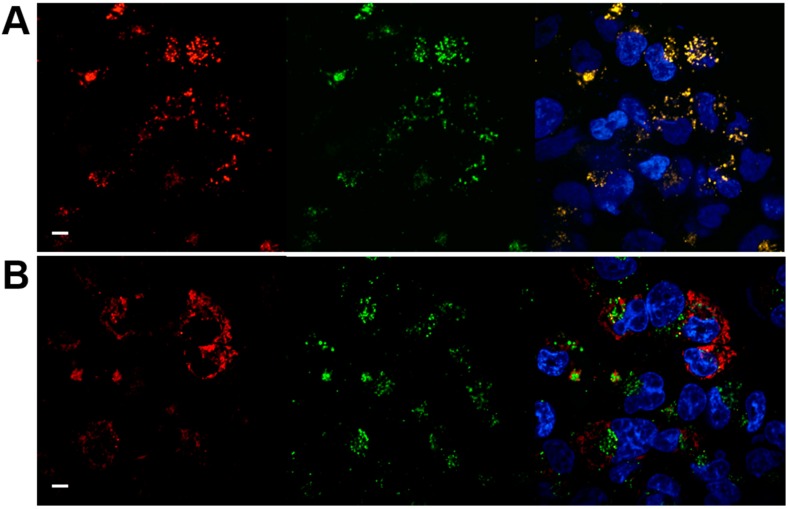

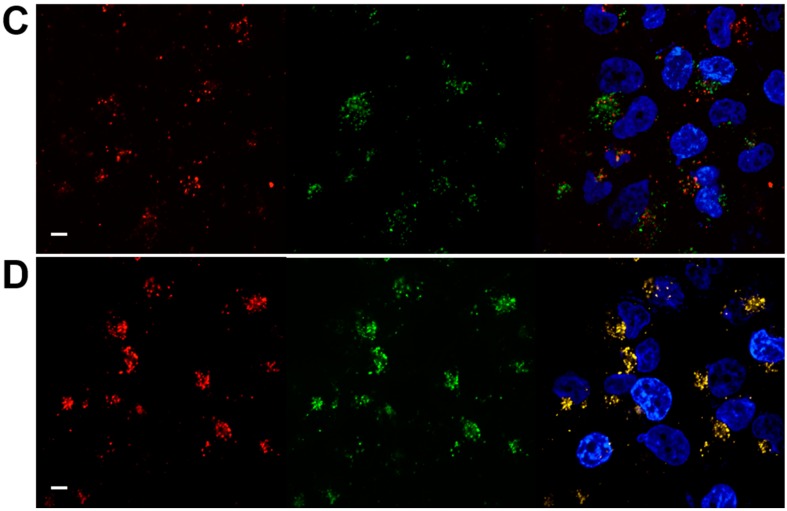
Representative images of intracellular distribution of Cy5-PHP and Cy5-TPGS in MCF-7/ADR cells from independently repeated experiments (*n* = 3). Cells were incubated with Cy5-PHP (**A**,**B**) and Cy5-TPGS (**C**,**D**) for 30 min (**A**,**C**) and 4 h (**B**,**D**) at 37 °C, followed by treatment with lysotracker for 30 min. The first column is the images of Cy-5 labeled PHP and TPGS (red) in the cells. The middle column is the images of lysotracker (green). The last column is the merged images of nuclei (blue), lysosomes (green) and Cy5-PHP (**A**, **B**)/Cy5-TPGS (**C**,**D**) (red). Scale bar = 11 µm.

Ideal nanocarriers can be rapidly endocytosed by tumor cells and quickly escape from lysosomes, allowing more entrapped drugs to enter the cell cytoplasm and reach the specific organelles. To study the intracellular trafficking of nanoparticles, MCF-7/ADR cells cocultured with Cy5-labeled PHP and TPGS micelles were observed by confocal microscopy. As shown in Figure 6, after incubation for 30 min, PHP micelles were found colocalized with lysosomes at a high rate (Pearson correlation coefficient, 0.93), suggesting the internalization of nanoparticles may undergo endocytosis pathway. However, after co-incubation for 4 h, the red fluorescence of PHP micelles was distinctly separated with the green fluorescence of lysosomes (Pearson correlation coefficient, 0.43), indicating the lysosomal escape. It is crucial for intracellular drug delivery that is capable of avoiding the degradation and exocytosis by lysosomes. On the other hand, the overlay of TPGS micelles with endo/lysosomes was increased over time (Pearson correlation coefficient, 0.54@30 min; 0.9@4 h), indicating TPGS held the weak ability of lysosomal escape.

### 3.6. Membrane Destabilization by PHP Copolymers

**Figure 7:**
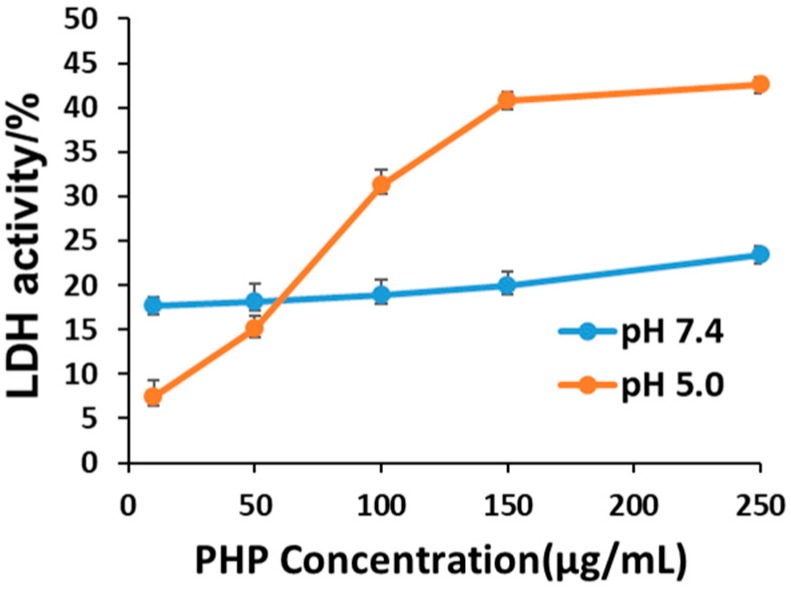
LDH liberated from MCF-7/ADR cells upon PHP at 37 °C for 1.5 h. Results were expressed as mean ± SEM (*n* = 6).

PHP copolymer is a typical polycation that has been extensively used for the non-viral gene delivery with a relatively high efficiency. In addition to the proton sponge effect, the membrane destabilizing capacity of PHP copolymer was further evaluated in MCF-7/ADR cells by LDH assay. Figure 7 clearly shows that PHP, as a pH-sensitive polymer, induced LDH release in a pH- and concentration-dependent manner. These data suggested that PHP might be able to facilitate the escape of endocytosed nanoparticles from lysosomes by membrane destabilization at relatively low pH.

### 3.7. Inhibition of P-gp Efflux

**Figure 8:**
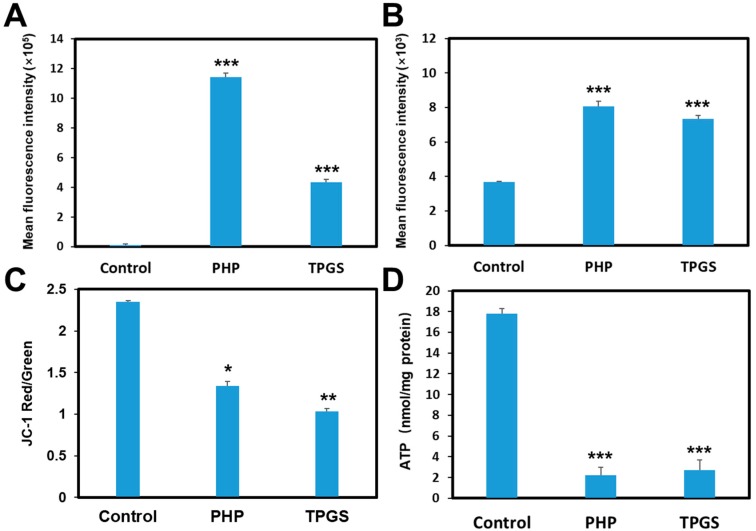
The cellular effects of PHP in MCF-7/ADR cells. The effects of different micelles on Rh123 accumulation (**A**); inhibition of Rh123 efflux (**B**); mitochondrial membrane potential (**C**); and intracellular ATP (**D**) were shown. The concentrations of PHP and TPGS were 250 µg/mL. Data as mean ± SD (*n* = 3). * *p* < 0.05, ** *p* < 0.01, *** *p* < 0.001.

To investigate the potential effect on P-gp efflux inhibition of PHP copolymeric micelles, Rh123 was used as a substrate of P-gp in MCF-7/ADR cells where P-gp was overexpressed. TPGS, a P-gp inhibitor, was used as a positive control. As seen in Figure 8A, Rh123 accumulation in PHP-treated cells was remarkably higher than that in untreated cells, as well as TPGS-treated cells. Similarly, the inhibition of Rh123 efflux by PHP was significantly higher than that of untreated control (Figure 8B). These results suggested that PHP might be able to inhibit drug efflux. Furthermore, the effects of PHP on mitochondrial membrane potentials and ATP levels were also explored. A decrease in the ratio of JC-1 red/green fluorescence indicates mitochondrial depolarization. As shown in Figure 8C, the ratios of JC-1 red/green fluorescence were significantly reduced by PHP or TPGS in MCF-7/ADR cells as compared to the control. In addition, PHP or TPGS also elicited a significant decrease in the intracellular ATP levels (Figure 8D). Mitochondrial membrane potentials are essential to maintain the physiological levels of the ATP and loss of mitochondrial membrane potentials reduces cell activity [28]. As P-gp is an ATP-dependent drug efflux pump, PHP-induced decrease in mitochondrial membrane potentials and ATP levels might account for the inhibition of P-gp-mediated drug efflux in MCF-7/ADR cells.

### 3.8. Cytotoxicity of DOX-Loaded PHP Micelles

**Figure 9:**
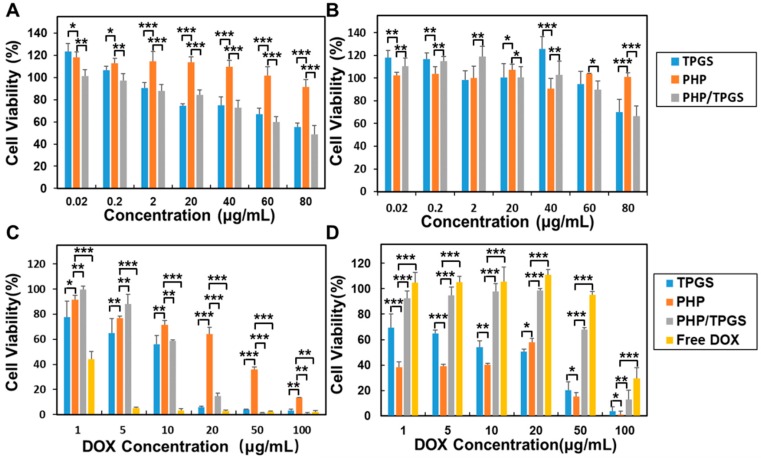
The cytotoxicity of MCF-7 (**A**) and MCF-7/ADR (**B**) cells incubated for 24 h with blank PHP, TPGS, or PHP:TPGS = 1:1 co-polymeric micelles as a function of polymer concentrations and the cytotoxicity of MCF-7 (**C**) and MCF-7/ADR (**D**) cells treated by free DOX, or DOX-loaded PHP, TPGS, PHP:TPGS = 1:1 co-polymeric micelles as a function of DOX concentrations. Data as mean ± SD (*n* = 6). * *p* < 0.05, ** *p* < 0.01, *** *p* < 0.001.

The cytotoxicity to MCF-7 and MCF-7/ADR cells of DOX-loaded micelles was examined using the CCK-8 assays. As shown in Figure 9A,B, blank PHP micelles showed minimal cytotoxicity in both cell lines, presumably because of the property of low charge density. Compared with DOX-loaded PHP micelles, DOX-loaded TPGS micelles exhibited higher cytotoxicity for MCF-7 cells (Figure 9C), because TPGS is a kind of surfactants that can induce cancer cell apoptosis [29]. The cytotoxicity of free DOX was dose-dependent and distinctly more effective in MCF-7 cells but less cytotoxic against MCF-7/ADR cells (Figure 9C,D). In Figure 5B, most of DOX accumulated in the nuclei of MCF-7 cells in free DOX group. By contrast, a significant portion of DOX was retained outside the nuclei in DOX-loaded PHP group. This might be because of slow release of free DOX from the nanocarriers.

DOX-loaded PHP micelles were more cytotoxic to MCF-7/ADR cells than free DOX (Figure 9D), suggesting PHP could overcome MDR effect in MCF-7/ADR cells. The IC_50_ of DOX-loaded PHP, TPGS and PHP/TPGS was estimated to be 7.89 µg/mL, 12.6 µg/mL and 53 µg/mL, respectively. Although PHP/TPGS could deliver slightly more DOX into MCF-7/ADR cells than TPGS (Figure 5C), DOX-loaded PHP/TPGS was less cytotoxic to MCF-7/ADR cells than DOX-loaded TPGS (Figure 9D). As PHP was protonated by low pH in lysosomes, positively charged PHP could interfere with the binding of negatively charged DNA to DOX, which consequently compromised the cytotoxicity of DOX. Although PHP moiety in TPGS/PHP could help deliver slightly more DOX into cells, it may not be sufficient to compensate the negative effect of PHP on DOX cytotoxicity in MCF-7/ADR cells. For PHP alone, it could deliver considerably more DOX into cells (Figure 5C), which overrode the negative effect of PHP (Figure 9D). Therefore, the mixture of PHP with TPGS might not be better than PHP or TPGS alone against MCF-7/ADR cells.

## 4. Discussion

In this study, a pH-sensitive micellar nanosystem was developed to deliver DOX into drug resistant breast cancer MCF-7/ADR cells. The results demonstrated that PHP could encapsulate DOX to form nanoparticles with uniform particle size and a good drug-loading capacity. DOX was released at a faster rate from PHP micelles in a weakly acidic environment (pH 6.5) than at pH 7.4. The pH sensitivity of PHP was tunable by changing the ratio of pH-sensitive moiety. Confocal microscopic observations showed quick cell-internalization and lysosomal escape.

In MDR cells, P-gp transporters can remove the drugs inside the cells. Therefore, previous studies have co-delivered chemotherapeutics and a broad range of P-gp modulators within one single nanoformulation to reverse the MDR [30,31,32]. Among them, various amphiphilic polymers with the superior bioavailability have been exploited to act as the promising macromolecular P-gp inhibitors, such as TPGS and pluronics [31,32]. TPGS is a water-soluble derivative of vitamin E with a lipophilic alkyl moiety and a hydrophilic polar moiety of polyethylene glycol 1000. TPGS has been widely used in preparation of various formulations as emulsifier, additive, stabilizer and permeation enhancer [33,34]. TPGS can presumably modulate P-gp efflux via the inhibition of P-gp ATPase [35]. A sort of TPGS-based copolymers, such as poly(lactide)-TPGS (PLA-TPGS), poly(lactide-co-glycolide)-TPGS, chitosan-TPGS and so forth, have been developed and exhibited superior ability against drug resistance [36,37]. For instance, Wang et al. [38] have synthesized a DOX-loaded copolymer containing ditocopherol PEG 2000 succinate, which has a combined effect of P-gp inhibition and antiproliferation. Li et al. [39] have also reported that TPGS-containing copolymer micelles could overcome the MDR in drug resistant MCF-7/ADR cells. Therefore, TPGS is a well-accepted agent to overcome the MDR in tumor cells.

In this study, we found that DOX-loaded PHP nanoparticles were more cytotoxic to MCF-7/ADR cells than DOX-loaded TPGS nanoparticles. Although TPGS is a well-studied MDR inhibitor, PHP-based drug delivery system was superior to TPGS against drug resistant cells, presumably because PHP, in addition to the inhibition of drug efflux, could enhance intracellular drug delivery. As shown in Figure 6, PHP could escape from endo/lysosomes. It is important to deliver DOX into the nuclei, as the main cellular targets of DOX are nucleic acids. By contrast, endo/lysosomal escape of TPGS was much less. Moreover, the inhibition of Rh123 efflux by PHP was also relatively more potent than that by TPGS (Figure 8B). Therefore, PHP could both enhance intracellular drug delivery and inhibit drug efflux, resulting in reversal of MDR effect in MCF-7/ADR cells. It remains elusive why DOX-loaded PHP/TPGS mixed polymers could not reverse MDR in MCF-7/ADR cells (Figure 9D). By contrast, these mixed polymers might be able to help deliver docetaxel into drug resistant A2780/T cells [40]. Difference in drug resistance mechanisms between cell lines may account for distinct effects of nanocarriers. According to our results, DOX-loaded PHP/TPGS mixed polymers may not be recommended to reverse MDR in MCF-7/ADR cells.

To further explore underlying mechanism how PHP could inhibit drug efflux, we examined mitochondrial membrane potentials and ATP levels in MCF-7/ADR cells. Similar to TPGS, PHP reduced mitochondrial membrane potentials and ATP levels in MCF-7/ADR cells. As P-gp is an ATP-dependent drug efflux pump, PHP-induced ATP depletion suppressed P-gp-mediated drug efflux. However, there was no significant difference in mitochondrial membrane potentials and ATP levels between PHP- and TPGS-treated cells, suggesting that PHP might inhibit drug efflux not only through suppressing P-gp. Further studies are required to fully understand the mechanisms underlying PHP-mediated drug efflux inhibition. Taken together, PHP polymeric micelles are able to effectively inhibit the proliferation of drug resistant cells. It warrants further investigation to validate the anti-MDR effect of PHP in mice. We envision that the combination of PHP with other tumor-targeting materials may further enhance the anticancer effect of chemotherapy against MDR.

## 5. Conclusions

We have developed a simple pH-sensitive drug delivery system. We found that this pH-responsive nanocarrier PHP could overcome the MDR in MCF-7/ADR cells by taking advantage of efficacious intracellular delivery of laden drugs and P-gp inhibition. Compared with TPGS, PHP may be a potentially superior tool for overcoming MDR.

## Supplementary Materials

The following are available online at http://www.mdpi.com/2079-4991/8/11/952/s1, Figure S1: Zeta potentials of PHP, TPGS and PHP/TPGS nanoparticles at different pH. Data are presented as mean ± SD (*n* = 6); Figure S2: Stability of nanoparticles in PBS, 2% BSA, 150 mM sodium chloride and cell culture media for three days at 37 °C. The particles were examined by dynamic light scattering. Data are presented as mean ± SD (*n* = 6); Figure S3: Cumulative DOX release profiles of DOX-loaded nanoparticles in cell culture media at different pH. Data are presented as mean ± SD (*n* = 3).
