# Supplementary material for: pH-Sensitive Poly(β-amino ester)s Nanocarriers Facilitate the Inhibition of Drug Resistance in Breast Cancer Cells
Source: Nanomaterials (Basel). 2018 Nov 19;8(11):952. doi: 10.3390/nano8110952 (PMC6267427; doi:10.3390/nano8110952)
Supplement: Supplementary file 1 [file nanomaterials-08-00952-s001.pdf]

Supporting Information

pH-sensitive poly(β-amino ester)s nanocarriers facilitate the inhibition of drug resistance in breast cancer cells

Mengxue Zhou[1,2,†], Xingcai Zhang[3,†], Jin Xie[1], Rongxiang Qi[1], Huiru Lu[1], Stefano Leporatti[4,*], Jun Chen[1,2,*] and Yi Hu[1,2,*]

[1]CAS Key Laboratory for Biomedical Effects of Nanomaterials and Nanosafety, Multidisciplinary Research Division, Institute of High Energy Physics, Chinese Academy of Sciences, Beijing 100049, China

[2]University of Chinese Academy of Sciences, Beijing 100049, China

[3]John A. Paulson School of Engineering and Applied Sciences, Harvard University, Cambridge, Massachusetts 02138, United States

[4] CNR Nanotec-Istituto di Nanotecnologia, Polo di Nanotecnologia, Lecce 73100, Italy

†These authors are co-first authors who contributed equally to this work.

*Correspondence: stefano.leporatti@nanotec.cnr.it, chenjun@ihep.ac.cn, and huyi@ihep.ac.cn.

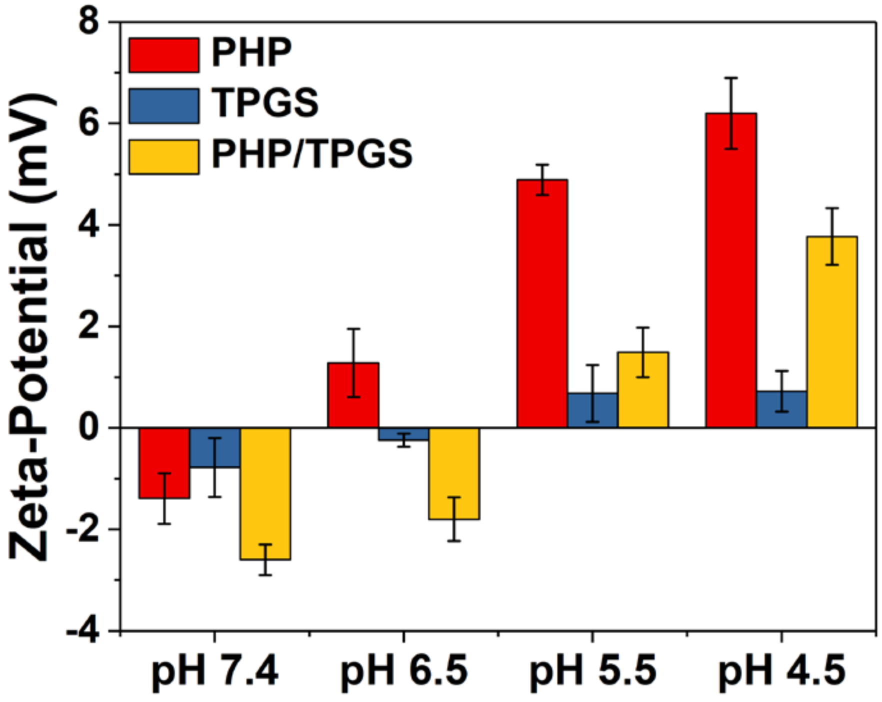

Figure S1. Zeta potentials of PHP, TPGS and PHP/TPGS nanoparticles at different pH. Data are presented as mean ± SD (n = 6).

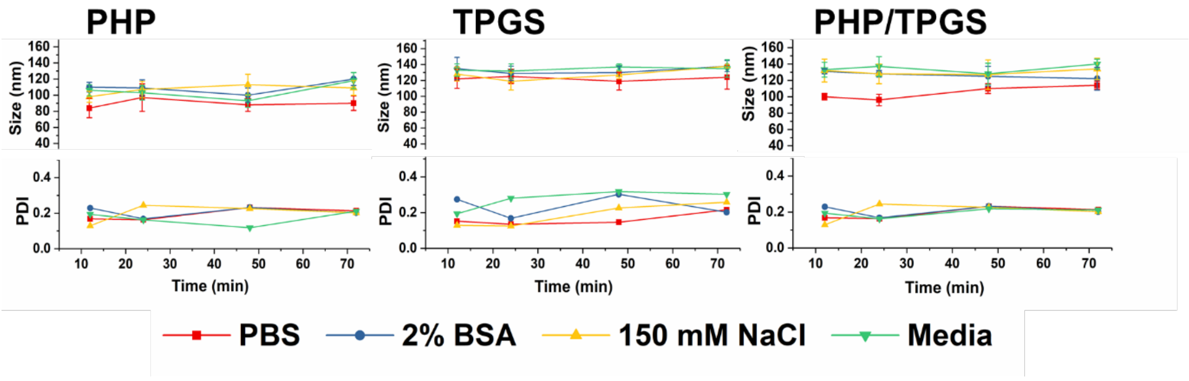

Figure S2. Stability of nanoparticles in PBS, 2% BSA, 150 mM sodium chloride and cell culture media for three days at 37 °C. The particles were examined by dynamic light scattering. Data are presented as mean ± SD (n = 6).

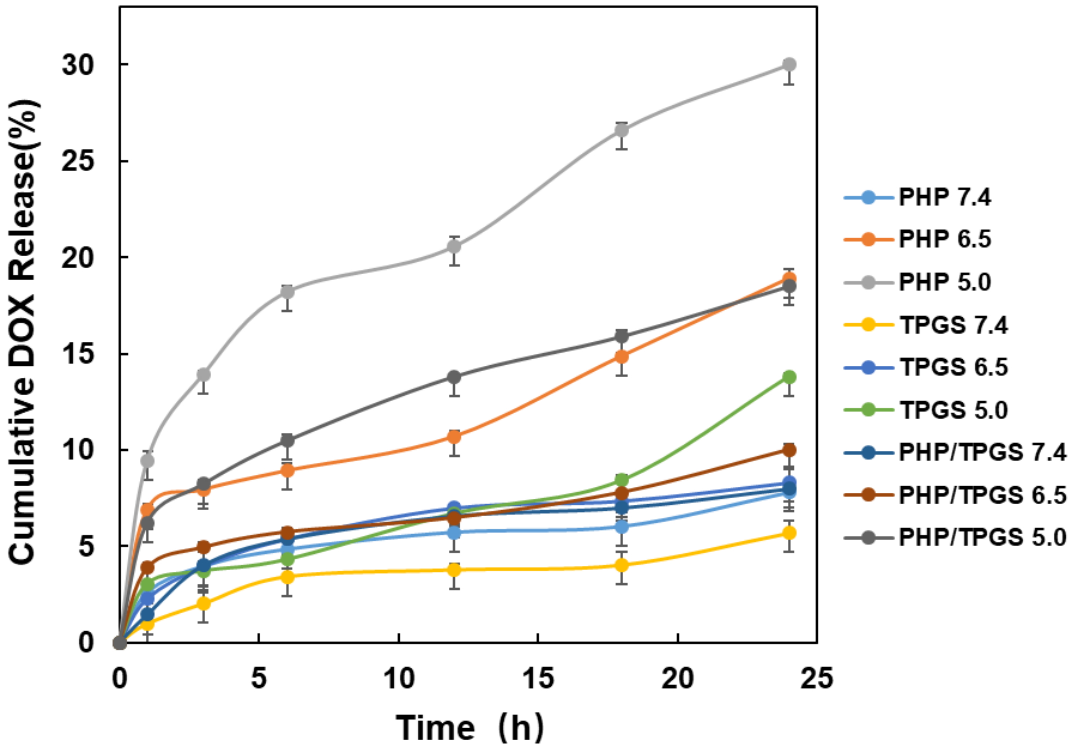

Figure S3. Cumulative DOX release profiles of DOX-loaded nanoparticles in cell culture media at different pH. Data are presented as mean ± SD (n = 3).
